# Supplementary material for: Structural characterization of the ICOS/ICOS-L immune complex reveals high molecular mimicry by therapeutic antibodies
Source: Nat Commun. 2020 Oct 8;11:5066. doi: 10.1038/s41467-020-18828-4 (PMC7545189; doi:10.1038/s41467-020-18828-4)
Supplement: Supplementary file 1 — Supplementary Information [file 41467_2020_18828_MOESM1_ESM.pdf]

Supplementary Information

Structural characterization of the ICOS/ICOS-L immune complex reveals high molecular mimicry by therapeutic antibodies

Rujas *et al.*

Supplementary Figure 1

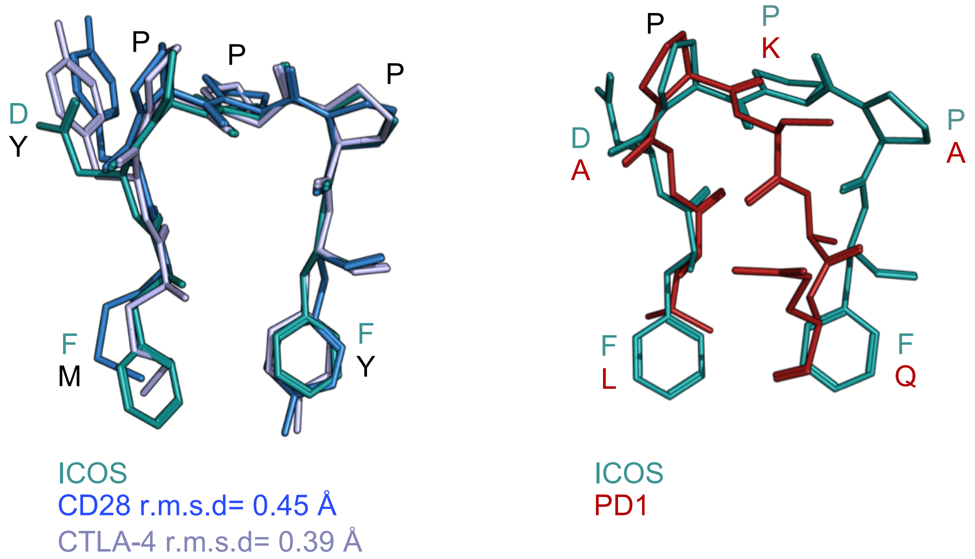

Structural conservation of the ICOS FDPPPF motif across family members. Superposition of the FDPPPF sequence of human ICOS (this study, deep teal), the MYPPPY sequence of human CTLA-4 (PDB ID: 1I8I, light blue), the MYPPPY sequence of human CD28 (PDB ID: 1YJD, blue) and the LHPKAK sequence of human PD1 (PDB ID: 4ZQK, dark red).

Supplementary Figure 2

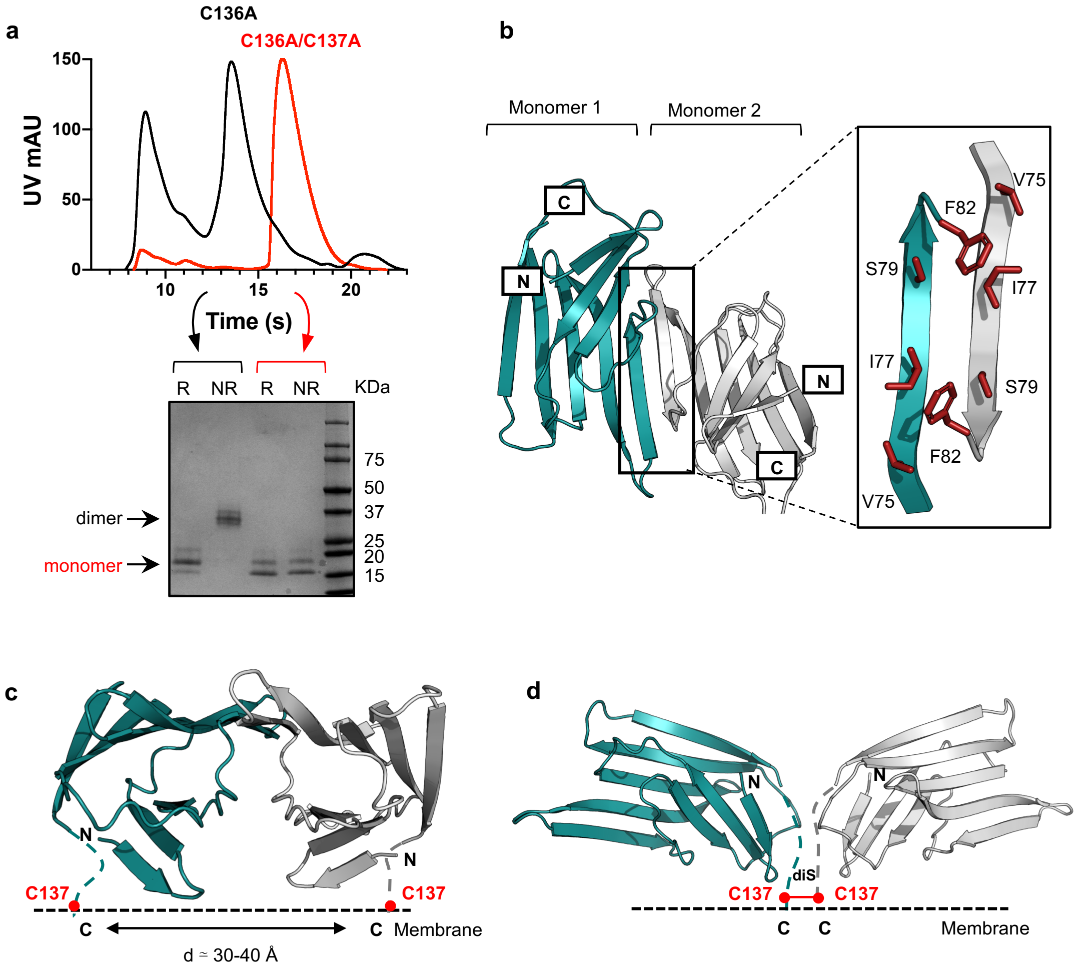

ICOS ectodomain dimerization. **a,** SEC profile and SDS-PAGE gels of ICOS ectodomain in the presence (C136A) and absence (C136A137A) of the interchain disulfide bond mediated by C137. NR, non-reducing; R, reducing. Dependency on C137 for the oligomeric state of ICOS was observed in more than three independent experiments. A representative case is shown. **b,** Ribbon diagram of ICOS dimer inferred from the crystal lattice placing the C-terminus of each monomer distal from one another. Close-up view of the residues involved in the crystallographic dimer interface. **c,** Membrane orientation of the crystallographic ICOS homodimer inferred from the crystal structure showing the distal position of C137 residues. **d,** Model proposed for the structural organization of ICOS homodimers at the membrane surface. The model was generated based on superposition of monomeric ICOS/ICOS-L complexes with the known CTLA-4 dimer (PDB ID: 1I8L).

Supplementary Figure 3

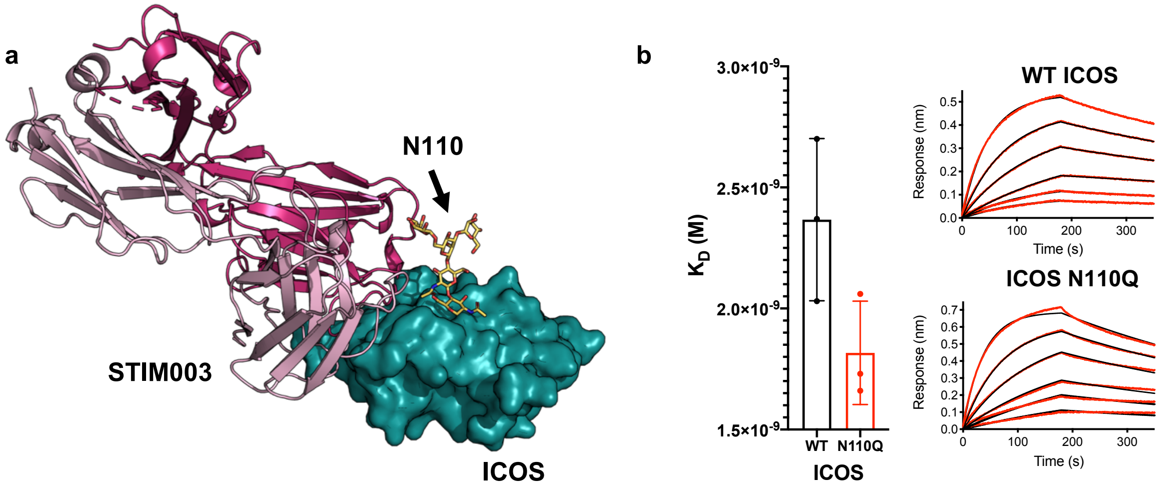

Effect of ICOS glycan N110 on STIM003 binding. **a,** Angle of approach of STIM003 binding (pink) to ICOS (deep teal). Glycan N110 is depicted in yellow. **b,** K_D and kinetic binding curves comparison of Fab STIM003 binding to ICOS in the presence and absence of N110 glycosylation. The mean values and standard deviation of three biological replicates are shown.

Supplementary Figure 4

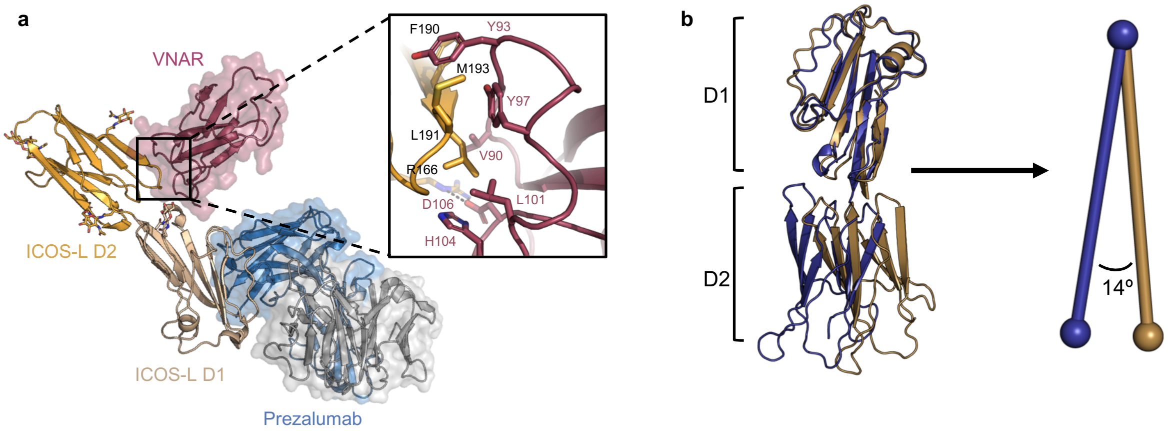

Antibody binding to ICOS-L. a) Binding of VNAR to the constant domain of ICOS-L. Inset: close-up view of the binding interface, which buries approximately 720 Å^2 of surface area on ICOS-L. Residues contributed by VNAR and ICOS-L are depicted in raspberry and orange, respectively. b) Relative orientation of the constant domain (D2) relative to the variable domain (D1) of ICOS-L when bound to ICOS (purple) and to prezalumab Fab (gold).

Supplementary Figure 5

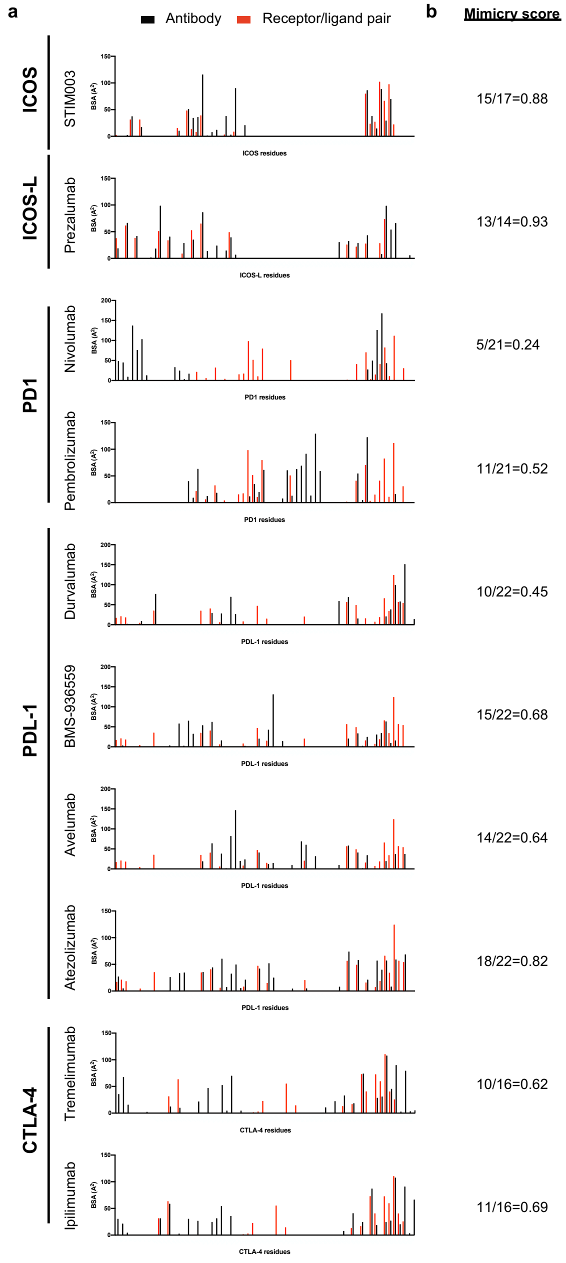

Antibody mimicry comparison. **a,** Comparison of residues contacted by the natural receptors and therapeutic antibodies targeting immune checkpoint receptors PD1, PDL-1 and CTLA-4. PDB entries used for the analysis: CTLA-4/B7-1 (1I8L), ipilimumab/CTLA-4 (5TRU), tremelimumab/CTLA-4 (5GGV), PD1/PDL-1 (4ZQK), pembrolizumab/PD1 (5JXE), nivolumab/PD1 (5WT9), atezolizumab/PDL-1 (5X8L), durvalumab/PDL-1 (5X8M), avelumab/PDL-1(5X8M), BMS-936559/PDL-1 (5GGT). **b,** Antibody mimicry scores calculated as indicated in **Fig. 6d**

Supplementary Figure 6

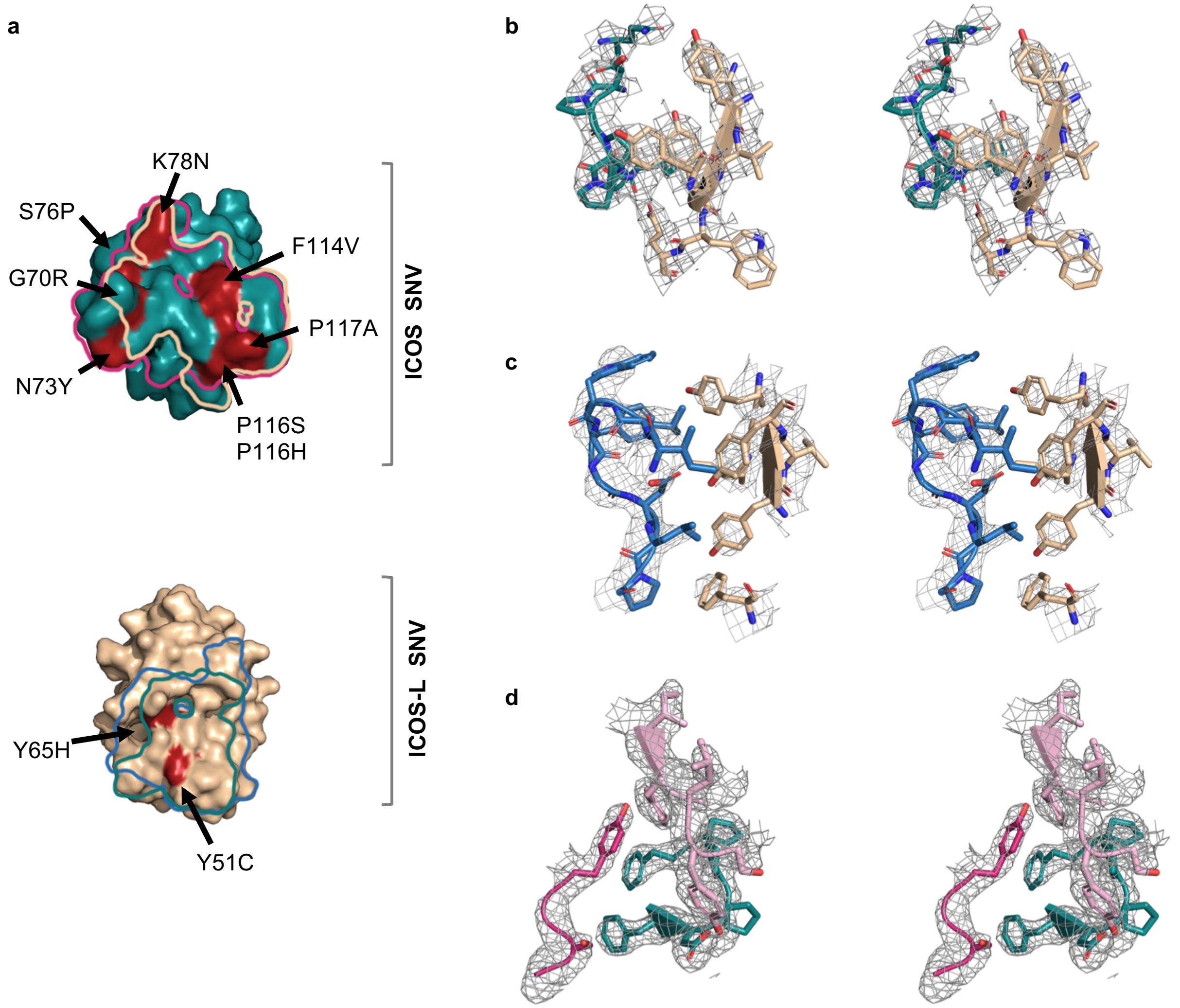

Binding interfaces of the three structures reported in this study. **a,** Single nucleotide variations (SNVs) identified in ICOS and ICOS-L interface in cancer patients as compiled from the BioMuta database[56]. SNVs are colored in red on the surface of ICOS and ICOS-L. Antibodies, receptor and ligand epitope traces are depicted for clarity. Stereo-image of the composite electron density contoured at 1.0 sigma at the interfaces of **b,** ICOS/ICOS-L, **c,** ICOS-L/Prezalumab and **d,** ICOS/STIM003. Color coding is as in **Fig. 4**.

Supplementary Table 1

Binding kinetics parameters.

Immobilized	Analyte	K_{on} ($M^{-1}s^{-1}$)	K_{off} (s^{-1})	KD (nM)
ICOS	ICOSL	$2.3\times10^5 \pm 3.5\times10^4$	$1.6\times10^{-1} \pm 1.3\times10^{-2}$	722 ± 89
ICOS-L	$ICOS_{dimer}$	$4.3\times10^5 \pm 9.2\times10^4$	$1.2\times10^{-3} \pm 7.1\times10^{-4}$	3.0 ± 0.6
ICOS-L	$ICOS_{dimer}$ F114A	n.b.	n.b.	n.b.
ICOS-L	$ICOS_{dimer}$ Q50A	n.b.	n.b.	n.b.
ICOS-L	$ICOS_{dimer}$ F119A	n.b.	n.b.	n.b.
ICOS-L	$ICOS_{dimer}$ N110Q	$9.3\times10^5 \pm 5.3\times10^4$	$7.1\times10^{-4} \pm 4.2\times10^{-5}$	0.7 ± 0.7
STIM003	$ICOS_{dimer}$	$3.0\times10^5 \pm 4.5\times10^4$	$7.2\times10^{-4} \pm 1.1\times10^{-4}$	2 ± 3
ICOS-L	Prezalumab	$8.2\times10^5 \pm 9.1\times10^4$	$4.5\times10^{-3} \pm 2.0\times10^{-4}$	5.5 ± 0.8
STIM003	$ICOS_{dimer}$ N110Q	$5.6\times10^5 \pm 7.7\times10^4$	$1.0\times10^{-3} \pm 7.7\times10^{-5}$	1.8 ± 0.2
